# The RAG Model: A New Paradigm for Genetic Risk Stratification in Multiple Myeloma

**DOI:** 10.1155/2014/526568

**Published:** 2014-09-11

**Authors:** Steven M. Prideaux, Emma Conway O'Brien, Timothy J. Chevassut

**Affiliations:** Brighton and Sussex Medical School, Sussex University, Falmer, Brighton BN1 9PS, UK

## Abstract

Molecular studies have shown that multiple myeloma is a highly genetically heterogonous disease which may manifest itself as any number of diverse subtypes each with variable clinicopathological features and outcomes. Given this genetic heterogeneity, a universal approach to treatment of myeloma is unlikely to be successful for all patients and instead we should strive for the goal of personalised therapy using rationally informed targeted strategies. Current DNA sequencing technologies allow for whole genome and exome analysis of patient myeloma samples that yield vast amounts of genetic data and provide a mutational overview of the disease. However, the clinical utility of this information currently lags far behind the sequencing technology which is increasingly being incorporated into clinical practice. This paper attempts to address this shortcoming by proposing a novel genetically based “traffic-light” risk stratification system for myeloma, termed the RAG (Red, Amber, Green) model, which represents a simplified concept of how complex genetic data may be compressed into an aggregate risk score. The model aims to incorporate all known clinically important trisomies, translocations, and mutations in myeloma and utilise these to produce a score between 1.0 and 3.0 that can be incorporated into diagnostic, prognostic, and treatment algorithms for the patient.

## 1. Introduction

Molecular studies have made it apparent that multiple myeloma is not a single disease entity but rather a collection of genetically diverse disease subtypes that manifest clinically as the clonal proliferation of plasma cells. With this, it is clear that a universal treatment approach is not sufficient and that patient management should be targeted towards the specific genetic disease subtype(s) a patient harbours. To fully achieve this, along with the somewhat established approaches of conventional cytogenetics and fluorescent* in situ* hybridisation (FISH), the myeloma genome from each individual will likely require sequence analysis as part of a standardized approach, with an algorithm/model then existing to elucidate clinically valuable meanings from the findings. This paper therefore proposes a concept myeloma “traffic light” risk stratification model, named the RAG (Red-Amber-Green) model, which aims to simplify data produced from genetic analysis into an accessible and intuitive form to inform disease risk stratification in the clinical setting. As a concept, the RAG model is designed to begin the process of attempting to integrate complex genetic information, particularly derived from sequencing technologies, into a simple risk stratification system.

## 2. Challenge of Designing Genetic Risk Stratification Models

Despite a consensus within the field that the integration of genetic information into the diagnosis, treatment, and prognosis of myeloma would be of great benefit, there is currently no universally accepted system to achieve this. The main challenge in designing such a model for clinical use is that often an abundance of complex data must be simplified into an intuitive and useful form whilst remaining valid and applicable. This balance is difficult to obtain, as models which are too complex become clinically unintelligible whereas those which are too simple lose accuracy and informative power. As a variety of molecular techniques are available to analyse the myeloma genome, a proposed genetic based model must be able to either incorporate the findings from a range of methods or be specifically designed to unite one. For reasons discussed hereafter, the RAG model is designed to accommodate multiple analytical methods but crucially expands on other risk stratification systems by attempting to accommodate whole genome sequencing (WGS)/whole exome sequencing (WES) data, as although these techniques are currently still highly experimental and cannot currently be used to accurately inform treatment decisions or risk/prognosis, it appears likely that due to their power and increasing accessibility these techniques will play a key role in the workup of myeloma patients in the future. Furthermore, although the RAG model is presented here as a concept for risk stratification, the benefits of which are to optimize outcomes and stratify treatment regimes in order to minimize toxicity, it is possible that in the future such a model will inform many different areas of myeloma medicine such as identifying new disease biomarkers and therapeutic targets and helping better genetically categorise/diagnose myeloma disease subtypes. The advances that these areas would bring to myeloma treatment alongside risk stratification make it even more pressing for models to be developed which accurately interpret and utilise genetic information.

## 3. Current Methods of Myeloma Risk Stratification and Prognostication

To establish a context for the requirement for a genetic risk stratification model, such as the RAG model, a literature review covering current myeloma risk stratification and prognostication follows.

### 3.1. International Staging System for Myeloma

For any malignancy to have a simple, accurate, and easily applicable universal staging system to inform prognosis is of obvious benefit. The first such staging system for myeloma, developed by Durie and Salmon in 1975 [1], used standard laboratory measurements and imaging to predict patient outcomes. This system was based mainly on measuring tumour burden and although still a useful means for achieving this today with improving treatments and extended patient survival the system proves less reliable for modern day prognosis. Subsequent to the Durie and Salmon model, the importance of the prognostic factors of serum *β*_2_-microglobulin (S*β*_2 _M) and serum albumin, which reflect disease burden plus renal function and patient “fitness,” respectively, emerged and led to the development of the international staging system (ISS) for myeloma (Table 1) [2]. The ISS is extremely convenient to use and proves applicable for prognostication across the majority of myeloma treatment settings. However, with the increasing use of molecular techniques to analyse the myeloma genome, there is a necessity for the integration of genetic information into the ISS to contemporise the system, especially as the system is recognised as being limited in power to detect the very highest-risk patients. With this, the RAG model is presented here as a starting point, not to supplant the ISS, but to put forward a concept on how the system may be supplemented through the integration of genetic information.

### 3.2. Myeloma Risk Stratification via FISH

With the ISS proving that measurements of disease burden, renal function, and patient “fitness” can accurately guide myeloma prognosis, a number of studies postulated whether FISH could be used to similar effect. In 2009, the International Myeloma Working Group (IMWG) evaluated the data published on the role of FISH in myeloma prognostication to formulate a consensus [3]. From their findings, the IMWG recommended that at a bare minimum a FISH panel testing for t(4;14), t(14;16), and del(17p) should be sought for all patients at diagnosis as these aberrations are associated with impaired survival and can genetically define a patient with high-risk disease. Since this recommendation, additional data has suggested that FISH analysis for +1q and t(14;20) should also be considered as these lesions would provide a more comprehensive assessment and further aid risk stratification [4]. As myeloma is often associated with gross chromosomal abnormalities, such as trisomies and translocations [5], the above recommendations state that conventional karyotyping should be combined with FISH analysis where possible as the technique provides quantitative chromosomal information which will supplement the more subtle structural changes detected by FISH. Despite this, if only one technique is employed, it is recommended that FISH be chosen due to the more specific and clinically useful information provided [6].

Recently, an important study conducted by Boyd et al. used outcome data from the Medical Research Council (MRC) myeloma IX trial to conduct multivariate analysis on the interaction of genetic aberrations identified via a comprehensive FISH panel [4]. Based on their findings, the genetic lesions associated with both short progression free survival (PFS) and overall survival (OS) included +1q21, del(17p13), and an adverse IGH@ translocation group incorporating t(4;14), t(14;16), and t(14;20). The study found that these adverse lesions often cosegregated and that an association between the accumulation of these lesions and worsened PFS and OS existed. The study then developed a novel risk stratification system by allocating patients into high-, intermediate-, and favourable-risk groups based on their FISH characterisation. The favourable-risk group was defined by an absence of +1q, del(17p13), and adverse IGH@ translocations, the intermediate-risk group harbours just one of these adverse lesions, and the high-risk group is defined by the cosegregation of >1 adverse lesion. This grouping proved accurate for prognostication and was independent of the ISS. The observation within this study that an accumulation of genetic lesions worsens outcome suggests that no one adverse finding on its own can define a high-risk group and implies that if FISH was to be used for risk stratification, a clearly defined and comprehensive panel would be required.

### 3.3. Combining FISH and the ISS for Myeloma Risk Stratification

As the ISS considers different factors to FISH, several groups have attempted to combine these two methods to adjudge whether a more optimal prognostic model can be developed. These studies are important as any model utilising genetic data for prognosis will ultimately require some consideration of tumour burden and host “fitness” to optimise accuracy. Boyd et al., using their initial risk stratification groups from the MRC IX trial, integrated the ISS and identified that an ultrahigh-risk group could be defined at diagnosis by ISS stages II or III plus >1 of their defined adverse lesions [4]. This ultrahigh-risk group constituted 13.8% of patients in the study and strongly predicted a poor outcome with median PFS and OS at 9.9 months and 19.4 months, respectively. Similarly, in a French study conducted via the Intergroupe Francophone du Myélome, 1064 young (<66) myeloma patients enrolled in homogenous therapeutic trials were prognostically assessed via FISH analysis [7]. Integration of their findings with the ISS revealed that a high-risk group consisting of t(4;14) or del(17) lesions and a high S*β*_2 _M accurately predicted short survival to a greater degree than the ISS alone. Providing similar evidence, retrospective analysis of 2642 patients comprehensively analysed using FISH from the IMWG database showed that combining both t(4;14) and del(17p) along with the ISS significantly improved PFS and OS prognostication [8]. Furthermore, recent data analysed from three separate myeloma trials has shown that patients with t(4;14) and/or del(17p) in addition to ISS stage III and/or high levels of lactate dehydrogenase (LDH) are at high risk of progression-related death despite modern treatment strategies [9]. These findings demonstrate that the prognostic accuracy of the ISS can be enhanced through the incorporation of both biochemical and genetic parameters.

### 3.4. Limitations to FISH in Myeloma Risk Stratification

Despite the contribution FISH analysis has made to improving myeloma understanding, a particular limitation to the technique is that it may only detect predefined genetic lesions determined by the specific probes employed. As the majority of key myeloma FISH lesions are already likely defined, this limitation is unlikely to prevent a well-constructed FISH library from becoming part of a universally accepted prognostic/risk stratification system. However, this limitation does prevent FISH from being utilised as a parallel screening tool to identify unknown genetic aberrations across the whole genome, an attribute which sequencing technology possesses and one which would improve therapeutic development and biological understanding. This point is well demonstrated by WGS recently identifying previously unobserved* BRAF* mutations in seven out of 161 (4%) myeloma patients screened with the technique [10]. These patients harbouring* BRAF* mutations may therefore benefit from treatment with newly developed* BRAF* inhibitors, drugs that in some instances have shown marked clinical activity [11]. Further to this limitation, some patients with “poor” prognostic FISH lesions have shown very good survival [7, 12, 13], bringing the specificity of these FISH findings in certain instances somewhat into question. Due to this, it appears the application of FISH to define risk needs refinement and/or supplementation as if current methods were solely relied upon there is the potential risk of patient harm through either over- or undertreating with chemotherapy.

### 3.5. Myeloma Risk Stratification via GEP

As FISH proved useful in myeloma prognostication, studies began to assess whether gene expression profiling (GEP) could also be used effectively. Shaughnessy et al. first assessed 532 newly diagnosed myeloma patients with GEP and, using log-rank tests of expression quartiles, identified 70 genes which were linked to shorter durations of remission, event-free survival (EFS), and OS [14]. Interestingly, they found that 30% of these genes were mapped to chromosome 1, with the majority of upregulated genes on 1q and downregulated genes on 1p. Using a ratio of mean upregulated to downregulated gene expression, a high-risk score was defined which proved to be an independent predictor of outcome endpoints in multivariate analysis that included the ISS. To further their work, multivariate discriminate analysis was performed which revealed that a 17-gene subset of the original 70 genes could predict outcomes as well as their original findings. The study therefore concluded that GEP could be used to accurately define high-risk disease through a genetic signature, a feature which may guide future therapeutic interventions by identifying over- or underexpressed genes/pathways.

A second study conducted by Decaux et al. investigated the GEP of 182 myeloma patients and identified the 15 strongest genes associated with length of survival [15]. These genes were then used to formulate a risk score which stratified patients into a high-risk group, characterised by the overexpression of genes involved in the cell cycle and cell cycle surveillance, and a low-risk group, characterised by a heterogeneous GEP pattern with a concomitant hyperdiploid signature. To validate the model, it was first tested in a set of 68 patients and then secondly in three independent myeloma cohorts totalling 853 patients. The kaplan-Meier estimates of OS at three years for the low- and high-risk groups were 90.5% and 47.4%, respectively, and were independent of traditional prognostic factors such as those of the ISS. This study again demonstrated that the genetic signature of myeloma could be used to predict survival.

### 3.6. Limitations to GEP in Myeloma Risk Stratification

Interestingly, of the 17 and 15 genes identified by the two aforementioned studies, none were shared, a finding which demonstrates the genetic complexity of myeloma. Furthermore, in these GEP studies, as for FISH analysis, the gene signatures used to define high-risk disease were not always specific for a given clinical outcome, a problem which may again lead to potential over- or undertreatment with chemotherapy. Additionally, gene mapping arrays only analyse the genetic signature of the predominant clone, whereas WGS can provide semiquantitative analysis of the size of the clonal population carrying a given aberration allowing for characterisation of disease substructure [16]. Furthermore, GEP is also required to be combined with FISH analysis to detect certain important prognostic lesions, as some, for example, del(17p), cannot be assayed by the technique. From the cumulative information on the use of GEP, it is clear that the technique has the potential to contribute towards myeloma prognosis. However, the technique is still largely experimental and not widely available and several issues require addressing before this technique becomes a practical option.

### 3.7. mSMART Prognostic Factors and Risk Stratification Model

The mayo stratification of myeloma and risk-adapted therapy model (mSMART) is a set of consensus guidelines developed by over 20 Mayo clinic myeloma physicians which aims to provide recommendations for the treatment of myeloma patients [6]. Two key aspects to the guidelines, relevant to this paper, exist. Firstly, the mSMART model has collated together the multiple validated biological factors which influence prognosis, and therefore risk, and classified them into 3 groups: tumour biology, tumour burden, and patient related factors. From within the tumour biology category, which is formulated through the use of conventional cytogenetics, FISH and GEP, a risk stratification system has been composed placing patients into a high-, standard-, or intermediate-risk group (Table 2) [6]. The mSMART group recommend that all myeloma patients undergo cytogenetic testing and risk stratification at diagnosis as these groups have been validated across multiple centres and correlate with a median OS of 3 years for high-risk, 4-5 years for intermediate-risk, and 8–10 years for standard-risk disease [17–21]. These risk groups can therefore importantly help determine not only prognosis but also the intensity and length of treatment regimes.

## 4. The RAG Model

As is evident, the inclusion of genetic information into a myeloma risk stratification system is both required and achievable at a given level. The biggest obstacle to progression in this area is how best to interpret and use the large amounts of complex data that the aforementioned molecular techniques generate. Answers to this problem must soon be developed as the accessibility, speed, and cost of the techniques will soon mean they are available for utilisation in the workup of a newly diagnosed myeloma patient. The following subsection therefore outlines a proposed genetic risk stratification model, developed as a concept, to try and create a starting point for how genetic information, particularly sequencing data, may be utilised.

### 4.1. RAG Model Categorisation System

Many groups have produced different biological classification systems for myeloma developed through a range of molecular techniques [7, 22–24]. For the RAG model, a new classification system is not proposed, but what is proposed is a novel categorisation method to collate genetic aberrations under relevant groups.  Table 3 demonstrates the categories proposed and identifies whether these represent an initiation/primary or progression/secondary event, an important distinction as the initiating/primary events contribute significantly towards disease behaviour, as demonstrated by the difference between hyperdiploidy and nonhyperdiploidy subtypes [5, 25, 26], whereas progression/secondary events can initiate transition to a more aggressive disease state and/or promote drug resistance [5]. It is recognised that the categories for the progression/secondary events are highly generalized; this however is purposeful and is designed to promote simplicity and interpretation, features that are essential if a genetic model is to breech the gap between genetic research and the clinical environment.

### 4.2. Candidate Genes for the RAG Model

Using conventional cytogenetic and FISH techniques, it has long been recognised that gross structural and numerical chromosomal changes are important in defining myeloma risk. With the recent employment of GEP and WGS/WES, however, the important genes in these instances are being identified whilst other salient genes, disrupted through more subtle structural changes/mutations, are becoming apparent. With this, the RAG model aims to expand on current systems of risk stratification by incorporating individually mutated genes deemed to be important in driving myeloma pathogenesis. For this, the genes relevant to the categories outlined in Table 3 are now listed in Table 4. It should be stated that in the model certain genes, for example,* MMSET*, are listed twice, as in these instances the gene may be both disrupted through a gross karyotypic structural/numerical aberration or be independently mutated and therefore relevant to another category. This addition allows the model to be fully inclusive by recognising that genes may be disrupted through multiple mechanisms.

### 4.3. Calculating a RAG Score

**Figure 1:**
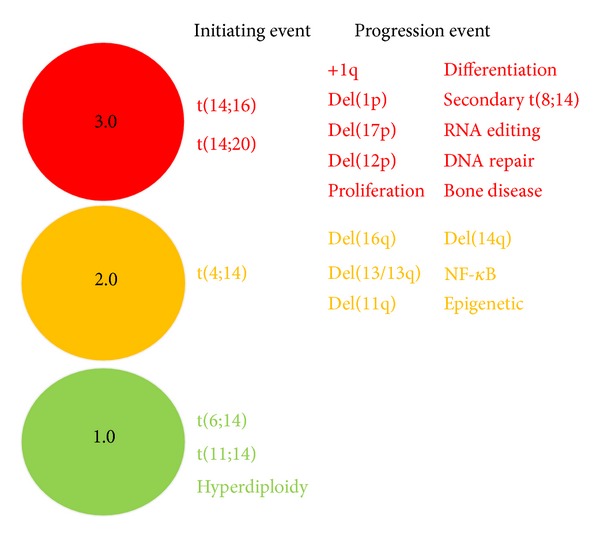
The RAG model. The genes and categories selected for the RAG model are placed into their respective red, amber, and green groups. To generate a RAG score, the average score for lesions correlating between a patient sample and the model is calculated. The RAG score is then used for risk stratification.

The model for calculating a RAG score is outlined in Figure 1, and, from this, it is apparent that the aberrations categorised in Tables 3 and 4 are now stratified into a red group, worth an arbitrary value of 3.0, an amber group worth 2.0, and a green group worth 1.0. By inputting the corresponding genetic aberrations from a patient sample, a mean RAG score can be calculated for risk stratification. Using this system, it is proposed that four stratified groups can be formulated. Those scoring 1 to 1.5 are deemed low-risk, those scoring 1.5 to 2.0 are low-intermediate-risk, those scoring 2.0 to 2.5 are a high-intermediate-risk, and those scoring 2.5–3.0 are high-risk (Table 5). This score is proposed as a pragmatic means of stratifying the aggressiveness of myeloma between a restrictive range of 1.0 and 3.0 based on genetic findings. A mean method rather than a summative method has been chosen as it is technique-independent and puts greater weight towards the more important genetic aberrations as outlined in the model. This is as opposed to a summative method which would be technique-dependent, insofar as the more extensively you analyse for, and detect, aberrations the higher a score you would produce. It should be stated that the score devised is relative to myeloma as a disease, where it is known that even small degrees of genetic abnormality can result in an aggressive phenotype and poor survival. Therefore, the low-, intermediate-, and high-risk groups should be viewed in light of typical myeloma survival times. A particular mention is required for t(4;14), as historically this has been considered a high-risk feature. However, with the implementation of newer agents, especially the proteasome inhibitor bortezomib, these patients are demonstrating far greater survival [20, 27], and so it is proposed here that the translocation be considered an intermediate-risk lesion.

### 4.4. Selection of RAG Model Categories and Genes

To determine the selected genes and categories to compile the RAG model, the evidence from studies linking genetic aberrations to prognosis/risk was reviewed. The criteria used to evaluate the evidence was based on established methods used by other groups and is represented in Table 6 [28]. For a gene to have been selected, it must have been independently supported by grade A recommendation, with those aberrations linked to a poor prognosis being placed into the red group, those linked to superior survival placed into the green group, and those of a neutral prognostic impact placed into the amber group. This method was applied where possible; however, certain genes, especially within the NF-*κ*B and epigenetic groups, are included as they are deemed important due to functional characterisation and mutational recurrence, as supported by grade A recommendation; yet studies specifically assessing their independent link to prognosis have not been conducted. This may compromise a degree of accuracy at present, though studies investigating these genes independently are likely to become available soon, and as the RAG model is designed to be a concept model, it is certainly adaptable and will require modification in the future where appropriate.

### 4.5. Application of the RAG Model

It is envisaged that cost and availability permitting, a model such as the RAG model will be used both at diagnosis and at later disease stages/relapse to build a genetic pattern over time. As it is recognised that myeloma progresses in union with an ever-changing genetic landscape through the advancement and regression of clonal tides [29, 30], it appears likely that a patient's RAG score will alter in line with genetic changes. This change in risk score, and newly identified aberrations, would be accepted within the remit of the model's application and may help direct adjustments to therapies and/or treatment intensities for patients along with restratifying risk. In further reference to therapeutic guidance, it should be stated that a patient's RAG score will be interpreted alongside the administered treatment regime as this may allow patterns of genetic abnormalities to be identified which could assist in determining resistance patterns. Furthermore, taking account of disease progression whilst on treatment and a short duration of response are recognised as therapy-related high-risk factors which may represent additional nongenetic factors to consider for prognosis [31, 32].

### 4.6. RAG “Pizza” Plots

**Figure 2:**
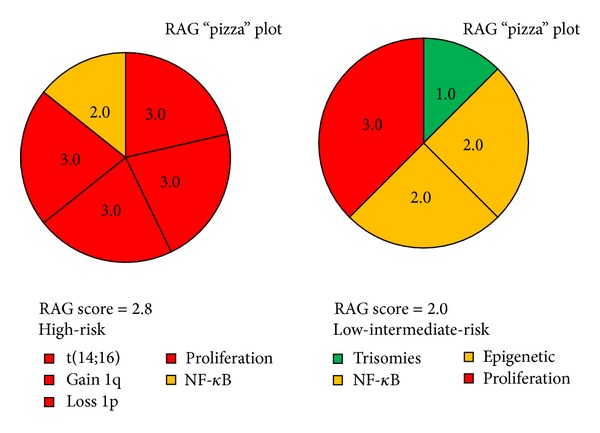
RAG “pizza” plots. The RAG “pizza” plots are colour plots which represent how the RAG score will be presented. The size of the segment each aberration represents is proportional to the “weighting” that lesion is given in calculating the RAG score; that is, a red group lesion will be represented by a larger segment when compared to the segments of amber and green lesions. Representation of the RAG score as a “pizza” plot helps to visualize the score and improve understanding.

To help visualize the RAG score and facilitate a better understanding of its meaning in the clinical environment, it is proposed that the score will be represented as a RAG “pizza” plot, whereby the genetic categories are displayed as colour plots as outlined in Figure 2. As a greater “weight” is given to the higher-risk lesions, these represent larger segments of the plot in comparison to lower-risk lesions. It is proposed that a more detailed report would be made alongside the RAG “pizza” plot for each patient outlining the specific genes and mutations present within the genome. Providing information on the specific mutations is likely to have particular importance for treatment decisions, especially in the future, as targeted therapies against specific aberrations are likely to be available and will facilitate an era of personalized cancer treatment.

### 4.7. Limitations to the RAG Model

It is recognised that by simply stating candidate genes within the RAG model that the system fails to outline the specific mutations relevant within that gene. This is important, as the functional consequence of two different mutations within a gene may well be different. Furthermore, it is recognised that synonymous mutations may occur within genes and therefore impart no alteration to protein structure. However, in a WGS/WES study by Chapman et al., [10] a nonsynonymous/synonymous ratio of 39 : 0 was observed in significantly mutated genes and it is therefore predicted that through the RAG model only including pathogenically important and recurrently mutated genes that the frequency of silent mutations will be low. Despite this, it is accepted that an algorithm, perhaps implemented alongside genomic reporting, will be required to demonstrate the functional importance of individual mutations. Furthermore, it is accepted that the model does not perhaps accurately reflect how genetic aberrations interact* in vivo*, as by using a mean method the model does imply that a low-risk mutation may ameliorate some of the influence of a high-risk lesion to the risk outcome, a factor which seems unintuitive but is at present unknown. Lastly, and most importantly, it is recognised that the RAG model is yet to be clinically validated and that the proposed model has been compiled by collating and extrapolating the information from a range of studies each with varying designs and limitations. It is however predicted that suitable data sets, that is, those using a range of molecular techniques including WGS/WES against survival, will soon become available and will provide the opportunity to clinically validate the RAG model and allow conclusions to be made on the accuracy of the system.

## 5. Conclusion

The RAG model is presented here as a concept for myeloma risk stratification system based on the genetic characteristics of the disease. The justification for the need of such a model is apparent from reviewing the literature which demonstrates that the underlying genetic makeup of an individual's disease contributes significantly towards behaviour and outcome and can be used to inform risk. The RAG model has been designed to begin the process of bridging the gap between experimental research and clinical medicine whereby there is a necessity to simplify large amounts of complex data into a useful and formative structure. Although principally designed to inform disease risk, it is envisaged that in the future a model such as the RAG model would be able to also contribute towards the identification of new disease biomarkers and therapeutic targets and aid in the genetic categorisation and diagnosis of myeloma disease subtypes.

In conclusion, despite its lack of clinical validation and optimisation, we believe that the RAG model has enormous potential to simplify otherwise complex genetic data to guide clinicians and improve treatment outcomes for patients. For this reason, we wish to present the model as a prototypic concept of how state-of-the-art genomic data can complement other established technologies to usher in an era of personalized myeloma medicine.

## Figures and Tables

**Table 1:** The international staging system for myeloma [2].

Stage	Criteria	Median survival (months)
I	Serum β_2_-microglobulin <3.5 mg/L and serum albumin ≥3.5 g/dL	62

II	Neither stage I or III∗	44

III	Serum β_2_-microglobulin ≥5.5 mg/L	29

*There are two categories for stage II: serum *β*_2_-microglobulin <3.5 mg/L but serum albumin <3.5 g/dL; or serum *β*_2_-microglobulin 3.5 to <5.5 mg/L irrespective of the serum albumin level.

**Table 2:** The mSMART risk stratification system in active myeloma [6].

High risk	Intermediate risk	Standard risk
FISH	FISH	FISH
del(17p)	t(4;14)	t(4;14)
t(14;16)		t(6;14)
t(14;20)		
GEP	Cytogenetic del(13)	All other patients
High-risk signature		
	Hypodiploidy	
	Plasma cell labelling index ≥3%	

**Table 3:** RAG model categories.

RAG category	Initiation/primary event	Progression/secondary event
Hyperdiploidy (Trisomies—1, 3, 5, 7, 9, 11, 15, 19, 21)	*✓*	
t(4;14)	*✓*	
t(6;14)	*✓*	
t(11;14)	*✓*	
t(14;16)	*✓*	
t(14;20)	*✓*	
+1q		*✓*
Del(1p)		*✓*
Del(11q)		*✓*
Del(12p)		*✓*
Del(13/13q)		*✓*
Del(14q)		*✓*
Del(16q)		*✓*
Del(17p)		*✓*
Secondary t(8;14)		*✓*
Bone disease		*✓*
Proliferation		*✓*
Apoptosis and NF-κB		*✓*
Differentiation		*✓*
DNA repair		*✓*
RNA editing		*✓*
Epigenetic		*✓*

**Table 4:** RAG model categories and their candidate genes.

RAG category	Candidate genes	Reference
Hyperdiploidy (Trisomies—1, 3, 5, 7, 9, 11, 15, 19, 21)	*CCND1, CCND2, CCND3 *	[22, 25]
t(4;14)	*FGFR3, MMSET *	[20, 23, 24, 27, 33]
t(6;14)	*CCND3 *	[7]
t(11;14)	*CCND1 *	[7]
t(14;16)	*c-MAF *	[24, 34, 35]
t(14;20)	*MAFB *	[21]
+1q	*CKS1B, ANP32E *	[4, 14, 36–39]
Del(1p)	*CDKN2C, FAF1, FAM46C *	[36, 37, 40, 41]
Del(11q)	*BIRC2, BIRC3 *	[36, 42, 43]
Del(12p)	*CD27 *	[12, 36]
Del(13/13q)	*RB1, DIS3 *	[7, 44–47]
Del(14q)	*TRAF3 *	[36, 42, 43]
Del(16q)	*CYLD, WWOX *	[36, 42, 43]
Del(17p)	*TP53 *	[7, 24, 48]
Secondary t(8;14)	*MYC *	[49, 50]
Bone disease	*DKK1, FRZB *	[51, 52]
Proliferation	*NRAS, KRAS, BRAF, MYC *	[10, 53]
NF-*κ*B	*TRAF2, TRAF3, CYLD, NFKB1, NFKB2, NIK, TACI, LTBR, BIRC2, BIRC3, WWOX, CD40 *	[10, 42, 43]
Differentiation	*XBP1, BLIMP1, IRF4 *	[10, 54]
DNA repair	*TP53, PARP1 *	[24, 48, 55]
RNA editing	*DIS3, FAM46C, LRRK2 *	[10, 36, 40]
Epigenetic	*KDM6A, KDM6B, MLL, MMSET, HOXA9 *	[10, 56, 57]

**Table 5:** RAG scores and their risk stratification groups.

RAG score	RISK Stratification
2.5–3.0∗	High-risk∗
2.0–2.5∗∗	High-intermediate-risk∗∗
1.5–2.0∗∗	Low-intermediate-risk∗∗
1.0–1.5∗∗∗	Low-risk∗∗∗

*Red color. **Amber color. ***Green color.

**Table 6:** Classification system for levels of evidence and grades of recommendation [28].

Type of evidence	
Level I—meta-analysis of multiple, well-designed, controlled studies. Randomized studies with low type 1 and type 2 errors (high power) are also considered.	
Level II—evidence obtained from at least one, well-designed experimental study. Randomised trials with high type 1 and/or type 2 errors (low power) are also considered.	
Level III—well-designed, quasiexperimental studies such as nonrandomised, controlled single-group, prepost, cohort, time, or matched case-control series.	
Level IV—well-designed, nonexperimental studies, such as comparative and correlational descriptive and case studies.	
Level V—case reports and clinical examples.	

Grade of recommendation	

Grade A—evidence of level I or consistent findings from multiple levels II, III, and IV studies.	
Grade B—evidence of levels II, III, or IV with generally consistent findings.	
Grade C—evidence of levels II, III, or IV but findings are inconsistent.	
Grade D—minimal or no systematic empirical evidence.	
